# Annotations

**Published:** 1902-04-05

**Authors:** 


### Annotations.

### The Persistence of Enteric Fever in the Army.

Sir Walter Foster recently drew the attention of the House of Commons to the continued prevalence of enteric fever among our troops in South Africa. He very properly insisted that the wastage from this cause was not taken sufficiently seriously, and we cannot but feel that Mr. Brodrick's reply to his remarks, so far from rebutting actually confirmed the charge which he brought against the sanitary organisation. Sir Walter Foster stated that during the months of January and February of the present year there had been 800 deaths from enteric fever among the troops—a mortality heavy in itself and representing a drain on our forces by no means creditable to the administration—and he definitely asked whether anything was being done in the way of providing the columns with the means of boiling water. To this question no answer seems to have been vouchsafed—an omission which can only be taken as implying a most serious neglect of the costly lessons which we have received during this war—and all that Mr. Brodrick could do was to repeat the old assertion that soldiers on the march cannot be controlled. He is reported as saying that "soldiers tired and thirsty would drink water whenever they came across it. A column arrived at a place and, while food was being prepared, the men in defiance of regulations made off to the river and drank the water." Of course they do if no other water is available. We do not believe, however, that the discipline of our troops is so bad that they would habitually act contrary to the regulations by drinking unsafe water if safe water were provided. As Sir Walter Foster says, this question is not taken "sufficiently seriously." If it were we should not see the matter regarded with such helpless fatalism; suggestions for improvement would not be met with the *non possumus* which is now looked upon as a sufficient negation to every proposal; and such an answer as that given by Mr. Brodrick could never have been offered by a responsible Secretary of State.

### The Reorganisation of the Army Medical Service.

It is impossible to put everything into a Royal Warrant, so we must not carp at the new Army Order because it does not contain every provision which one might have wished to find within it. We are still left entirely in the dark as to whether it is intended to provide a sufficient number of officers to do the work which has to be performed, nor does the Warrant contain any promise of reform in regard to that long-standing grievance as to Indian pay, which has done so much to shake all faith in Royal Warrants. It would therefore be going far beyond what is justified by the wording of the new Army Order to say that it will remove all the causes of dissatisfaction in the Royal Army Medical Corps. We may say at once, however, that practically every point which it touches it touches with an improving hand. In view of the provisions of the new Warrant, the Army Medical Service should prove attractive to many suitable men. First, there is the generally improved position which is sure to permeate to all ranks from the improved status secured to the Director-General, who is now placed on an equal footing with the heads of departments on the headquarters staff. Next we have the fact that instead of an army candidate having to go back again to his anatomy and physiology and pass an examination in elementary subjects, his entrance examination will in future be confined to clinical medicine and surgery. Then if a man is holding a post, say, as house surgeon to a hospital, when he passes his entrance examination he may be seconded for the period during which he continues to hold such appointment, while in any case the time he may spend at Netley will be reckoned towards his promotion. The examinations to which he will be subjected during his career seem sufficient to insure efficiency while they are not such as need deter any. The pay although not high ought to prove sufficient, while in regard to retirement the certainty of a pension of £1 a day after 20 years of service, which has always proved so important an attraction, has been preserved. Moreover, under the new arrangements there are many provisions whereby a man may obtain some recognition, by way of both pay and promotion, for good work done, so that taking it all in all the new Royal Warrant ought, we think, to prove very satisfactory to the profession.

### Cholera at Mecca.

For some years past little has been heard of cholera, for although it has been prevalent enough, and more than enough in its accustomed haunts, it has not extended much beyond them. This year, however, the occurrence of cholera at Mecca and Medina and at the port of Jeddah, has served to remind us again of the time when the presence of cholera in the Hedjaz used to be regarded as a direct menace to the safety of Europe. Much, however, has happened since then, more especially the general substitution of "medical inspection" for quarantine in the old sense of the term, and although a certain amount of discredit has no doubt fallen on the modern form of medical inspection, from the fact that it has largely failed in Eastern countries in the case of plague, it must be remembered that the whole discussion which led up to the Dresden Convention had reference not to plague but to cholera, so that we may fairly hope that the measures therein accepted by the various Powers will be found effectual in controlling the spread of this disease. There are circumstances, however, in regard to the present outbreak which cannot but cause some anxiety, more especially the fact that the disease broke out among certain pilgrims from Russia. Notwithstanding the repeated assertion that Europe is threatened whenever cholera appears in the Red Sea littoral, the really great epidemics have seemed to reach us by way of Russia. It is worth noting then that while the pilgrimage to Mecca has this year been more numerously attended than for several years past, the increase has been largely due to the number of Russian inhabitants of Central Asia who have taken advantage of the withdrawal of the embargo which has for some time been laid upon the pilgrimage so far as Russian subjects were concerned. Pilgrims returning by sea will no doubt be fully inspected, and every care will be taken to prevent the conveyance of infection. But the great caravans which return by the land towards Syria and the Caucasus are much more difficult to control.

